# Strategies to Locate Lost Persons with Dementia: A Case Study of Ontario First Responders

**DOI:** 10.1155/2021/5572764

**Published:** 2021-05-15

**Authors:** N. A. Neubauer, A. Miguel-Cruz, L. Liu

**Affiliations:** ^1^School of Public Health and Health System, Faculty of Health, University of Waterloo, Waterloo, Ontario, Canada; ^2^Department of Occupational Therapy, Faculty of Rehabilitation Medicine, University of Alberta, Edmonton, Alberta, Canada; ^3^Glenrose Rehabilitation Research,Innovation & Technology (GRRIT) Hub, Edmonton, Alberta, Canada

## Abstract

Information on strategies and practices in the search of missing persons with dementia is inconsistent which creates challenges for first responders, such as police, when they choose appropriate search and rescue approaches. The purpose of this study was to describe current strategies among police services in Ontario. Telephone interviews with police were conducted. Questions included what strategies were used for locating missing persons living with dementia, and what gaps exist in search practices. Participants described they used high- and low-tech solutions in search and rescue. They identified gaps in education and awareness, proactive strategies, resources, and funding. Information collected from the interviews was used to develop a practice guideline for police in partnership with the Alzheimer Society of Ontario.

## 1. Introduction

The rates of dementia are on the rise as populations age. More than 747,000 Canadians are living with dementia [1] with the prevalence expecting to more than double every 5 years [2]. One significant concern is when persons with dementia become lost and go missing, especially when they are alone or in unfamiliar environments [3]. The adverse consequences of getting lost include high search and rescue costs [4], injuries [5], and death [6]. The average cost estimates of missing person investigations can range from $2,294 to $4,181 CAD [7], and if a person with dementia is not found within 24 hours, up to half will experience serious injury or even die [8].

It is estimated that approximately 40% of those diagnosed with dementia will become lost at some point in their disease progression [9]. Thus, searches that involve missing persons with dementia in Canada will increase with the rising national prevalence of dementia. It has been reported that missing persons with dementia typically continue to “go until they get stuck” [10] which differs from other vulnerable groups. As a result, search and rescue (SAR) strategies for persons with dementia may differ from other populations. Tracking or locator technologies, such as wearable global positioning systems (GPS) [11] and radio frequency identification (RFID) [12], for example, may offer options for mitigating risks while providing a person with dementia autonomy to go about safely outside of their home. Other strategies such as MedicAlert^®^ Connect Protect [13] and vulnerable persons registries [14] may also provide the information needed to police services in a timely manner to assist them in their search.

While alternative SAR strategies to address missing persons incidents involving individuals living with dementia have become increasingly available in recent years, the literature suggests that the information describing such strategies is limited and their overall scientific evidence is low [15]. In addition, SAR personnel may remain unaware of the behavioural differences that occur in persons with dementia who become lost [16]. As a result, police services may resort to using traditional SAR strategies. Such strategies may include the use of helicopters and tracking dogs instead of focusing on strategies specific to a missing person's anticipated behaviour, or the use of media releases to notify the public [15]. In addition to a higher success in locating a missing person with dementia, targeted strategies may also mitigate the costs of search and rescue for this population. The cost to operate a standard helicopter alone averages $1,600 US/hour [17].

The lessened awareness of dementia-related behaviours among SAR personnel could be attributed to a lack of available education materials and resources [15]. This can create challenges for police services to identify specific SAR strategies that are appropriate for locating missing persons with dementia. Thus, this study aimed to describe existing strategies and gaps that experienced among police services in Ontario, Canada, used to search for missing persons with dementia to help inform the development of a practice guideline for this population.

## 2. Methods

### 2.1. Participants

Participants for this study were a purposive sample recruited via word of mouth. One to two media officers and one to two officers with experience in SAR were recruited from each respective region of Ontario, Canada (North, East, Central, South Central, greater Toronto, and South West) (*n* = 30). All participants had experience in media releases or search and rescue of missing persons with dementia, were English speaking, and at least were 18 years of age. Ethical approval was obtained from the University of Alberta Research Ethics Board 2 (Pro0076365).

### 2.2. Data Collection and Analysis

A qualitative description [18] approach was used for this study. We used an inductive data collection approach and a constant comparative method [19]. One-time, semi-structured telephone interviews with individual participants were 60 minutes in duration and included 12 questions. Interview questions with police covered three main categories: (1) strategies related to SAR and media releases among missing persons with dementia; (2) tipping point related to the use of these strategies; and (3) strategy gaps.

All interviews were digitally recorded and transcribed verbatim. Transcripts and field notes were read and reviewed multiple times to ensure accuracy of codes assigned to short segments of text. We used Excel and NVivo to organize the data for analysis. We used directed content analysis [20] and developed an initial coding scheme based on an interview guide. We summarized and cross-referenced the data for each semi-structured interview in a table.

## 3. Results

### 3.1. Participant Demographics

The sample comprised police officers in SAR and media from 16 police services across Ontario (*n* = 27) (Table 1). Participants varied in types of experiences working with missing persons who have dementia. Among the police participants, experiences included SAR manager roles, vulnerable missing persons or emergency services coordinators, watch commander of an operations centre, duty inspector, involvement in ground searches, and media releases of missing persons to the public.

### 3.2. Search and Rescue Practices for Missing Persons with Dementia

Table 2 presents a total of 14 types of strategies described in the interviews with police in SAR. The most frequently reported strategies across all six regions in Ontario were search tactics (i.e., drones, dogs, helicopters) (100%), Project Lifesaver^®^ (40%), MedicAlert^®^ (40%), education and awareness (33%), and vulnerable person registries (33%). Participants reported that these strategies were typically learned through formal training (100%), resources such as SAR related textbooks and information made available from local Alzheimer Societies (13%), conventions or conferences (13%), and experiential learning (13%) (Table 3). Most interviewees referred to Robert Koester's book “Lost Person Behaviour” as their primary reference material for search tactics for missing persons with dementia [21]. In particular, they referred to a chapter from this book that describes how to determine the direction of travel of the person with dementia. According to this chapter, persons with dementia generally go until they get stuck. Based on this observation, SAR managers can expect to find this population in areas where other missing persons do not venture, such as near or in bodies of water, marshes, wooded areas, and bushes. Additional search tactics that were used within police services in Ontario include training members of the search to start looking for identification bracelets due to the raising implementation of strategies such as MedicAlert^®^. Depending on the resources available to each department, these strategies were either purchased or rented from vendors.

In terms of search tactics of SAR, police referred to search urgency policies that determine how quickly search teams need to be dispatched. Across all regions within Ontario, police prioritize missing persons with dementia as a highest urgency due to the missing persons' inability to care for themselves. Other methods above and beyond search tactics include education and awareness with long-term care and the public in terms of understanding dementia and the necessity to call the police as soon as someone with dementia is believed to be missing. Locating technologies such as Project Lifesaver^®^, identification methods such as MedicAlert^®^, alerting public transit personnel, and use of vulnerable persons registries were also discussed as proactive strategies that should be investigated and incorporated within police practices.

### 3.3. Practices for Media Release in Cases of Missing Persons with Dementia

For media releases, three categories of strategies were identified: (1) processes, (2) social media, and (3) disclosure about dementia (Table 4).

### 3.4. Processes

Most media officers spoke about the processes necessary for media releases of missing persons with dementia (Table 2). In general, the type of information released was reported to be specific to each individual incident. Picture, age, clothing, last known location, and medical condition were standard information across all cases. The end goal of a social media release was to get more eyes on the ground to keep a look out for the missing person. To engage in this practice, police highlighted the importance of getting these releases out to the public as soon as possible.

Search tactics across all services indicated that media releases were only drafted after police had been dispatched to the home and were able to confirm that the person is missing. Initial releases were sent off with vague information and follow-up releases were sent out with more detail as the investigation transpired. Processes were dependent on whether the police service was run through regional municipalities, or provincial. With regional services, for example, multiple media officers were trained on media releases. For some services, this has enabled officers to send these releases at home or work through mobile platforms.

“Yeah, and the neat thing when I was there is my BlackBerry was always on. Initially it took a while just to open up this new type of thought process but now your media officer is basically going to be at home or working and as soon as the missing person comes in and it's a quality-of-life issue, they're going to contact the media officer and say “Look, we want to get this out immediately” (media police officer 1).”

For other police services, drafted media releases are required to be sent to their portal first before it can be released to the public. This portal is then responsible for sending the release across the entire province to media outlets rather than to specific underlying regions.

“Whenever a media release is drafted up and ready to go out, I do not send it to them or any media outlet directly; it gets posted to what's called a provincial portal…From there it gets distributed. As soon as I hit the send button, it pretty much distributes right across the entire province to media outlets, you know, newspapers and radio stations and whatever–and they all have access to that portal (media police officer 2).”

### 3.5. Social Media

Social media was identified as the most common and effective method to alert the public of missing persons, with police resorting to Twitter, followed by Facebook as the main platforms. Mainstream media will then gain further information from these platforms and will release it through the news. The reason for this shift away from emphasizing newspapers and other traditional forms of media is because social media has the capacity for the releases to be immediate.

“I sent that out as a Tweet from our Twitter account–we've got like 10 000 followers which is pretty special. Thing about social media is to provide a meaningful and almost immediate opportunity for two-way communication between the police and the communities they serve. Twitter is preferred as has the fastest response and easy for headline searches due to 140 characters being available at a time. 99% of the time people are going to Tweet it because it's immediate and we can get that out as soon as possible (media police officer 2).”

Other services, however, still resort to using media outlets as their main strategy. This is because each individual detachment does not have access to social media, as they have only one larger regional twitter account. Due to the time sensitivity of these releases, for these services, local media is the fastest way they can get the information to the public.

“So, for us though, unlike some other police services, each individual station or detachment does not have social media, we have one larger regional twitter account. So yeah, for us, I mean without having the ability to post it ourselves, then a call or a post, you know. If it's super urgent, yeah, we have the local say radio station that we can call and say, can you post this on your Facebook page or can you Tweet this out and they'll do that on our ask (media police officer 4).”

### 3.6. Disclosure of Dementia

Identification of a missing person as having a cognitive impairment in these media releases was also brought up by media officers. In general, police thought if there was concern for the missing person's safety, including the term ‘dementia' in media releases can assist in generating sympathy from the public which may result in more eyes on the ground looking for the missing person. Police are obligated to gain consent from the family of the missing person as to what information can be released to the public, in order to respect people's privacy. With the stigma still evident around dementia, police services get varying responses, dictating whether they can disclose information that the missing person has dementia in their media releases.

“So, if the family says, no, I do not want that going out, there's nothing we can do, yeah, supposedly. We always encourage it [including ‘dementia' in media releases] (media police officer 1).”

“In circumstances where a family is against releasing that the missing person has dementia, police will phrase the release to the following in hopes of gaining attention from the public.”

“Police are very concerned for their wellbeing and anybody that has seen whomever, please contact police immediately (SAR police officer 5).”

Media and SAR officers thought that the use of media to alert the public was critical for rapid search of a missing person with dementia and its overall potential should not be ignored. Social media has the potential and recruit more eyes on the ground. It can inspire communities to dedicate resources, such as volunteers, to offer help in the search process.

### 3.7. Tipping Point for the Implementation of SAR and Media Release Strategies

The increased number of cases of missing persons with dementia was the tipping point for SAR to use social media strategies. One of the interviewees from the South-Central region commented:

“For us it was actually physically getting the increase of calls specifically towards whether they're elderly or they are diagnosed with dementia. Not all of them result in a full out search but the divisions they are dealing with, I'd almost say on a weekly basis where they have elderly people gone missing and whether it's just they've over extended themselves on a walk that they normally do or whether this is just the onset of some sort dementia that they have not been diagnosed with, and we're seeing it (SAR police officer 1).”

These findings were further supported by interviewees when asked questions regarding the collection of missing persons with dementia data. For example, across all Ontario regions, between 10 and 50% of missing persons calls in the last 5 years have involved a person with dementia, with increases in the incidents of these missing persons calls reported ranging from a 1% increase per year to the incidence doubling over the last 5 years (Table 5).

### 3.8. Gaps in Practice

Two general categories arose in terms of gaps in practice for the rapid search of missing persons with dementia among police services: (1) community education and awareness and (2) proactive strategies and available resources.

### 3.9. Community Education and Awareness

All participants highlighted that there was a misconception that care partners must wait 24 hours to call the police after a person with dementia goes missing. When an incident is reported later, the search radius is typically greater, further reducing their ability to locate missing persons in a timely manner.

“We also heard that, you know, “We are not allowed to call the police, unless it's been 24 hours with a missing person.” I do not know if that's just some misinformation, you know, that they hear, whether it's through the TV or not. They have this feeling there is a waiting period for missing persons where we try to say “Absolutely not, call us as soon as possible” because that just gives us a better time for us to get out there (SAR police officer 10).”

Another reason for this delay is a fear that care partners are wasting the police's time, resulting in families conducting their own ground search prior to initiating the call to police. One interviewee from the Central Region stated the following.

“The reluctance to call immediately, the feeling that with some people because especially with the older generation that they feel like they're wasting our time. I'm working in more of a rural division now up in our service we get that a lot. Like I literally was just dealing with a case this morning and all I got every second sentence from him was really sorry to waste your time. And you just reassure them that you know, this is where we want to be, we want to be involved early and often if we have to in order to stop the tragedy from happening (SAR police officer 5).”

In some situations, multiple missing occurrences took place before families contact police after they have not been able to locate the missing person after their own extensive searches. Care facilities were also known to delay contacting the police about a missing person with dementia.

Perhaps reporting, in the first instance, from care providers who do not necessarily see the gravity of the situation, call in a timely fashion. But there's nothing we're going to be able to do about that. You get the odd instance where somebody has waited–it's almost like they're–they've been caught with their hand in the cookie jar, and they're hoping to solve it themselves before calling (SAR police officer 12).

“In some circumstances, care facilities were unable to provide the information necessary for police to carry through with their investigation such as point of last seen, and what the missing person was wearing, which may contribute to the delay in reporting. Police suggested that care facilities need to keep records that contain the dementia resident's most recent photo, and clothing descriptions. One participant described a simple strategy for facilities to keep a binder on residents that is kept up to date.”

“So, what we find is the facilities are not–it's hard to say like it's not like they can keep track of all the of the patients 24/7 but at the same time you know we should be able to narrow it down within an hour or so. What we also see with the facility is that they are not able to provide us with clothing descriptions on a regular basis which means that they are not even sure what is in the person's closet–like any of those details are important for us. Several years ago, we tried to get an initiative going where all the facilities had a binder for each of their residences, especially those identified with dementia which was helpful (SAR police officer 3).”

Other education and awareness gaps include a lack of basic SAR training and education on dementia signs and symptoms for all officers, and the wider education to the public on how to determine whether an older adult with potential signs of dementia is missing. Interviewees emphasized that many individuals in the community, including some members of police services, are not aware that an older adult is lost. This is understandable because a person with dementia could look like any person. Education is significant to help with this and help people be aware of what signs to look for.

“In the end, people are more likely to pay attention to a lost dog. They're driving down the road and they see a lost dog and report it versus a random person that may be elderly. The assumption is that person wants to be there. There's not necessarily anything wrong. When I spoke with that woman [that was lost], my assumption was, “Okay, she just does not speak English and that's why she's confused.” When you're dealing with someone with dementia there are key things that set them aside from just someone who does not speak your language. I think that education component needs to get out there more so that people are more aware. A person could look dishevelled, dirty, elderly and confused and, “You know what, maybe they're just homeless. I do not really care.” They move on not realizing that person has been missing for four or five days, suffers from dementia and they're dirty because they've just been wandering the streets for the past five days. If I had known more then I probably would have contacted our police service. At least I could've got some attention there sooner, but it came down to simply not knowing and an assumption that she just does not speak English (SAR police officer 9).”

### 3.10. Proactive Strategies and Available Resources

Proactive strategies and available resources were another common gap noted by the interviewees. For most regions, there was a need to promote more proactive strategies such as vulnerable persons registries to assist police services in operating within existing department budgets.

“In terms of you know how we are going to go forward as a government how are we going to sustain long-term searches and you know and still operate within budgets that we have for policing services in our you know, respective jurisdictions. So, anything we can do to try and mitigate some of those expenses and also are going to be a benefit for each municipality and region in the province and so forth going forward (SAR police officer 6).”

Other gaps in available resources include the number of media and SAR officers that are available within certain jurisdictions. In one of the regions in northern Ontario, for example, there was only one media officer for the entire area. Therefore, when the officer was off duty, their area must be covered by a neighbouring police service, and vice versa. Within one of the other jurisdictions however, there was only one central twitter account from where media releases were sent. As stated by the police, this takes extra time, causing many detachments to resort to traditional modes of media rather than social media. This may have an influence on the level of effectiveness media releases can have for finding missing persons with dementia as soon as possible. This can also be seen with SAR where in some regions the closest SAR teams are more than an hour away.

“Our closest search and rescue is in London. So, our team is a 13-man team and they can hit the ground really quick and they can be effective, but obviously we're limited by numbers, right? So, to have a trained qualified large number search and rescue team here there would probably...by the time we decide that we want to activate them, get them to Sarnia, get coordinated, we're probably three to four hours before they're on route…so, yeah, that's probably our biggest gap, is getting them to go out in a timely way, but I do not know how to deal with that because we just do not have that volunteer base here (SAR police officer 2).”

While proactive strategies may reduce the time it takes to find missing persons with dementia and prevent future incidences, participants stated that everything comes down to costs. Strategies such as Project Lifesaver^®^ are expensive for police services to integrate, and locator devices may be associated with purchase and subscription costs to families, inhibiting some individuals from having access to these strategies.

“It's a subscription amount [locator devices and MedicAlert^®^], you know, and it's significant. So, it's difficult for us to, you know, we cannot push that on anybody. We can suggest it and obviously, it helps us significantly but the families maybe, you know, constrained for cost. Cellphones for us is a big thing, you know, if we can find out that person is carrying a cellphone and we'll reached out to local cellphone companies and do what's called a ping in order to try to triangulate a position where that person was or is. But again, not all people especially in that demographic are carrying cellphones. So, [Project] Lifesaver^®^ is something that is always at the back our minds, it's just something that is not feasible right now due to costs (SAR police officer 1).”

According to the participants, a means to subsidize proactive strategies is essential to ensure everyone has access to these resources and services.

“I think subsidy would be amazing because there are some families that cannot afford it or are not even aware of some these resources. And then the police services that we have, Senior support officers employed here at the service, that is a resource that at the front end that can push that information out. So that, you know, those tools can be utilized prior to somebody going missing. As much as we like, you know, we definitely would hand out these resources afterwards but let's try to stop it right from the front end and give out these resources so that, you know, we do not even come into the situation where one of these people have gone missing (SAR police officer 8).”

## 4. Discussion

The purpose of this study was to determine what existing strategies were being used by first responders in Ontario, Canada, that focused on missing persons with dementia. To our knowledge, this is the first study to examine and describe strategies used by police services within Canada to find missing persons with dementia.

In terms of existing practices among police, currently used strategies involved the use of search tactics, notifications to the public through media channels, vulnerable persons registries, and several strategies noted by (Neubauer et al.) [15], such as locator devices and identification methods. These strategies were used in part due to tipping points that were incurred by each police service, such as individual case studies, the influx in the number of cases and calls of missing persons with dementia, and the awareness of the urgency necessary to find this population due to their inability to survive for long on their own. While several strategies were already incorporated within police departments throughout Ontario, there were still gaps experienced by police when responding to and dealing with missing persons with dementia. One needed improvement would be to promote that one size does not fit all in terms of the adoption of police practices among varying police departments.

The types of strategies were also associated with the availability of resources. While Project Lifesaver^®^ was used within several police jurisdictions, others were unable to adopt it despite the high cost. We also do not know the effectiveness of these strategies [22]. This was beyond the scope of this project but would warrant a separate study; nevertheless, cost spending on strategies should be supported by evidence that the strategy or program is effective or makes a difference. The availability of police officers that can take on the role of media or SAR officer varied across jurisdictions, and chosen strategies depended on human resource capacity.

An issue identified by the interviewees was the common belief among the general public that care partners must not call the police immediately when a vulnerable older adult is missing. Participants explained that there was a significant time delay between when the person with dementia was last seen and when the police are notified. Suggested reasons were care partner's fear of wasting the police's time. In the case of a facility, staff would not report a missing person to the police immediately for fear of litigation and judgement. Some care partners may not appreciate the gravity of the situation until it is too late. These findings were also reported by Shalev Greene et al. [23], who described that the most common and important factors influencing the delay calling the police were caregiver's sense of embarrassment, guilt, and fear of disapproval or judgement by the police because they were unable to keep the person with dementia safe. Participants in this study also suggested that a fear of negative reactions by a person with dementia after finding out their care partner called the police, a caregiver's distrust of the police, and a desire to protect their relative were other reasons for not notifying the police immediately.

Older adults who are lost are not always recognizable by the public, nor by the police themselves. This was confirmed by the participants of this study. It is not uncommon for the public and police to mistake a lost person with dementia as someone who is either homeless or simply out engaging in regular activities, not requiring assistance. While topics of this nature underlying education and awareness have been brought forward within the literature [24–26], few resources are available within the grey literature, where police and communities can seek information of this nature [15]. The inclusion of awareness campaigns and publicly available education resources could be used to describe how missing incidents occur among persons with dementia. In turn, it could assist in informing the public why wandering behaviour is not always related to these occurrences [27], nor are they a result of poor caregiving [25]. This highlights the importance of alerting a community or reporting missing older adults and demonstrates the need for a greater emphasis and implementation of strategies and subsequent guidelines that can further address these education gaps [15].

The description of a missing person as having dementia or cognitive impairment was also inconsistently used in media releases. While the use of the term “dementia” in media releases resulted in more members of the public becoming engaged in keeping a lookout for the missing person, police were required to obtain consent from the missing person's family. According to the participants, it is common for family members to be against releasing this information, primarily due to the stigma associated with dementia and the potential of making the missing person even more vulnerable, such as people taking advantage of the missing persons who are described as vulnerable and cognitively impaired [28].

## 5. Conclusion and Recommendations

In conclusion, there is a variation of certain practices and strategies and there are also some consistent approaches in searching for missing persons with dementia. These strategies can be related to available resources. Evidence on the effectiveness of these strategies however remains limited. The development of a practice guideline of search strategies to find missing persons with dementia could reduce the time to locate these individuals and reduce the cost on police resources. In addition to the development of such a guideline, we propose the following recommendations to move the field forward:

1. Create education and awareness campaigns, and practice guidelines for police. Delays in the time it takes to call the police of a missing person with dementia and the ability to recognize whether a person with dementia is missing remains the largest most concerning issue for police. Stigma of dementia is a barrier to using the term dementia in media releases. There is general unawareness of the general public, including police, about whether an older adult is lost.
2. Create provincial or national vulnerable persons registries. This collection of information ahead of time for vulnerable older adults at risk would help to assist search and rescue personnel in timely media releases and searches. This strategy however will only work if all individuals with dementia register and it is free or affordable. Who would cover the costs and control the data would also need to be identified.
3. There is a need for research to verify effectiveness of strategies recommended by the participants to inform allocation of funding resources.
4. Establish consistent content and approaches to creating media releases through some of the police services, as well as the involvement of more trained SAR in all police services.
5. Identify search and rescue tools that have evidence of success and make these tools affordable. Participants recommended subsidization for proactive services and strategies such as locating technologies. While some police services have been able to fundraise money to assist in their costs, it still limits many organizations, families, and persons living with dementia to participate in some of these strategies, reducing the sheer potential these initiatives can have in terms of the rapid responses of finding this population as soon as possible.

## Figures and Tables

**Table 1:** Participant characteristics (*n* = 27).

Region in Ontario	Police officer type	Years of experience (years ± SD)	Age (years ± SD)	Gender
North (3); South West (4); South Central (5); Central (8); East (4); Toronto (2); Provincial (1)	SAR (17); media (10)	15 ± 7	45 ± 8	Male (21); female (6)

*Note.* SAR refers to police search and rescue, and media refers to police media. Each checkmark represents one participant.

**Table 2:** Strategies used by police for lost persons with dementia.

Strategy type	Total number of participants that used the strategy	Region(s) that used the strategy
Search tactics	15	North; South West; South Central; Central; East; Toronto; Provincial
Project lifesaver^®^	6	Central, North
MedicAlert^®^	6	South Central; Central, East; Toronto
Education and awareness	5	Central, South West
Vulnerable persons registry	5	South Central; Central, South West
Alerting public transit	4	South Central, Central, South West; Toronto
Media releases	2	North; South West; South Central; Central; East; Toronto; Provincial
Label clothing	1	East

**Table 3:** Modes of educating police on strategies for lost persons with dementia.

Education type	Total number of participants
Formal training	15
Conventions/conferences	2
Experiential learning	2
Resources	2

**Table 4:** Practices for sending an alert about a missing person on social media, by region.

Region in Ontario	Practice
North	Police orders stipulate what can and cannot be released to the public. Guidelines exist for drafting releases. Draft release is sent up chain of command to be reviewed. Depending on how serious the event is, release needs to go to headquarters for approval before it can be released

South West	Type of information through media release is dictated by each scenario. Officers are trained on what they can and cannot release. Will not release that the person has dementia unless this information plays a role in how to deal with the missing person

South central	Will double-check the place last seen before sending a release. Goes through chain of demand. By the time the media release comes to media officer, it is immediate. Information is screened before it gets to the media officers. Social media alert includes photo, clothing worn when last seen, where they were last seen, other descriptive information like height, and hair color. This information is deleted after the person is located. After the person has been found, the public is updated

Central	Do a search of the area. If not found, a picture of the person is obtained, age, last whereabouts, and other information. Social media releases do not say that missing person has dementia in order to respect individual's privacy. Alert does state that the police is concerned for the missing individual's wellbeing. From there, it goes to website, and is posted on Twitter and Facebook. Release can be posted in 10–15 minutes. At most, it has taken 30 minutes because family did not have a picture or were trying to look for a picture. Social media release is kept posted until person is found. After an extended period of time, police will post under missing persons page

East	Gathers the information from on-site investigators (i.e., picture, description of person, direction of travel). Send to Twitter, Instagram and Facebook, but Twitter is more effective. Time to media release depends on urgency of the investigation; if there is medical danger, it is sent out immediately. Weather is also a factor. Picture release is important as it clarifies identity of missing person. Every case is individual. If there is imminent danger, and depending on points they have, it gives authority to pursue further

Toronto	Information released is specific to individual incident. Approvals go through command and through investigation. If there is concern about criminality, then they need to involve Criminal Investigations Bureau. It is case specific but need to make sure the proper approvals are in place through the local area. Missing person must be verified by police officer. Call based on priority response which is influenced by weather and missing person's medical conditions. Once person is confirmed missing, then corporate communications is informed, and the police will report back to their station. Media relations will send photo, and gain authorisation. Social media alert will include information on dementia with permission of the family, so it gains sympathy from the public

*Note.* PWD refers to persons with dementia.

**Table 5:** Trend of calls about missing persons with dementia, by region.

Region in Ontario	Range (% of missing persons calls that involved persons with dementia)	Frequency	Trend over the last 5 years
North	30–50	N/A	Drastic increase
East	30–50	N/A	Steadily increasing
South Central	27–33	N/A	>1% increase per year
South West	10	N/A	Steadily increasing
Central	30–50	2–3 calls/12-hour shift	Increased. One department has doubled within the last 5 years
Toronto	N/A	5–7 calls/24 hours	Increased slightly

## Data Availability

Data are available upon request to the corresponding author.
